# Practice of traditional Chinese medicine for psycho-behavioral intervention improves quality of life in cancer patients: A systematic review and meta-analysis

**DOI:** 10.18632/oncotarget.5388

**Published:** 2015-10-15

**Authors:** Weiwei Tao, Xi Luo, Bai Cui, Dapeng Liang, Chunli Wang, Yangyang Duan, Xiaofen Li, Shiyu Zhou, Mingjie Zhao, Yi Li, Yumin He, Shaowu Wang, Keith W. Kelley, Ping Jiang, Quentin Liu

**Affiliations:** ^1^ Institute of Cancer Stem Cell, Cancer Center, Dalian Medical University, Dalian, China; ^2^ Sun Yat-sen University Cancer Center, State Key Laboratory of Oncology in South China, Collaborative Innovation Center of Cancer Medicine, China; ^3^ College of Nursing, Dalian Medical University, Dalian, China; ^4^ Department of Radiology, Second Affiliated Hospital, Dalian Medical University, Dalian, China; ^5^ School of Public Health, Dalian Medical University, Dalian, China; ^6^ Department of Psychology, Dalian Medical University, Dalian, China; ^7^ Dalian Medical University Magazine, Dalian, China; ^8^ School of Art, Dalian Medical University, Dalian, China; ^9^ Shanghai University of Traditional Chinese Medicine, Shanghai, China; ^10^ Integrative Immunology and Behavior Program, Department of Animal Sciences, College of ACES, Urbana, IL, USA; ^11^ Department of Pathology, College of Medicine, University of Illinois at Urbana-Champaign, Urbana, IL, USA; ^12^ Graduate School, Dalian Medical University, Dalian, China

**Keywords:** traditional chinese medicine, psycho-behavioral interventions, quality of life, cancer, meta-analysis

## Abstract

**Background:**

Cancer patients suffer from diverse symptoms, including depression, anxiety, pain, and fatigue and lower quality of life (QoL) during disease progression. This study aimed to evaluate the benefits of Traditional Chinese Medicine psycho-behavioral interventions (TCM PBIs) on improving QoL by meta-analysis.

**Methods:**

Electronic literature databases (PubMed, CNKI, VIP, and Wanfang) were searched for randomized, controlled trials conducted in China. The primary intervention was TCM PBIs. The main outcome was health-related QoL (HR QoL) post-treatment. We applied standard meta analytic techniques to analyze data from papers that reached acceptable criteria.

**Results:**

The six TCM PBIs analyzed were acupuncture, Chinese massage, Traditional Chinese Medicine five elements musical intervention (TCM FEMI), Traditional Chinese Medicine dietary supplement (TCM DS), Qigong and Tai Chi. Although both TCM PBIs and non-TCM PBIs reduced functional impairments in cancer patients and led to pain relief, depression remission, reduced time to flatulence following surgery and sleep improvement, TCM PBIs showed more beneficial effects as assessed by reducing both fatigue and gastrointestinal distress. In particular, acupuncture relieved fatigue, reduced diarrhea and decreased time to flatulence after surgery in cancer patients, while therapeutic Chinese massage reduced time to flatulence and time to peristaltic sound.

**Conclusion:**

These findings demonstrate the efficacy of TCM PBIs in improving QoL in cancer patients and establish that TCM PBIs represent beneficial adjunctive therapies for cancer patients.

## INTRODUCTION

Cancer patients experience a variety of adverse symptoms, including fatigue, anxiety, depression and pain, during the development and progression of disease. These psychological and physiological impairments reduce their quality of life (QoL). During the past two decades, there has been a movement away from emphasizing only pharmacological interventions to a more comprehensive approach in alleviating symptoms and improving QoL [1]. More specifically, psycho-behavioral interventions (PBIs) have been developed as beneficial coping strategies. Consisting of psychosocial, behavioral and physical therapeutic methods, PBIs consist of a series of non-pharmacological approaches, mainly utilizing stress management, cognitive-behavioral therapy (CBT) and physical training. The early focus of PBIs was primarily aimed at improving mental health [2]. The use of PBIs has now been extended to relieve adverse side effects of cancer chemotherapy through the use of relaxation, hypnosis and distraction [3–5]. With greater acceptance of PBIs in the medical community during the past decade, their utilization has increased rapidly and has now become a very promising approach for improving QoL in cancer patients [6].

Accumulating evidence supports the possibility that PBIs reduce stress and improve QoL, even in cancer patients [7, 8]. For example, psychosocial nurse counseling following conventional treatment is feasible and effective in reducing depressive symptoms in head and neck cancer patients [9]. In another stress management intervention, patients were randomly assigned to one group that received the standard of care and another group that received stress management training. Three weeks after the beginning of radiotherapy treatment, cancer patients with initial high psychological distress post-radiotherapy benefitted significantly from the stress management training [10].

Varies forms of complementary and alternative medicine have been accepted and practiced worldwide [11]. Complementary and alternative medicine includes many TCM PBIs, including acupuncture, Chinese therapeutic massage (tuina) and Tai Chi. All of these approaches which have garnered substantial interest by the medical community for their potential in improving QoL in cancer patients. Despite the growing acceptance of a variety of PBIs around the world, there is no comprehensive evaluation of high quality, well-controlled clinical studies on the use of TCM PBIs. Many individual TCM trials have reported conflicting results, and there is a real need for a comprehensive analysis of the potential benefits and safety of using TCM PBIs for nearly all diseases but particularly for cancer patients [12]. In this report, we employed a meta-analysis to determine the clinical effects of PBIs on QoL in 67 high-quality clinical trials involving 6,806 Chinese cancer patients. We found that PBIs improved QoL in these patients by improving sleep, reducing symptoms of depression and improving pain management, although there were subtle but real differences between symptom improvements caused by TCM and non-TCM PBIs. This new meta-analysis of controlled, high-quality clinical trials supports the conclusion that TCM PBIs benefits QoL in cancer patients by reducing a variety of adverse clinical symptoms with minimal safety concerns.

## MATERIALS AND METHODS

### Search strategies

**Figure 1:**
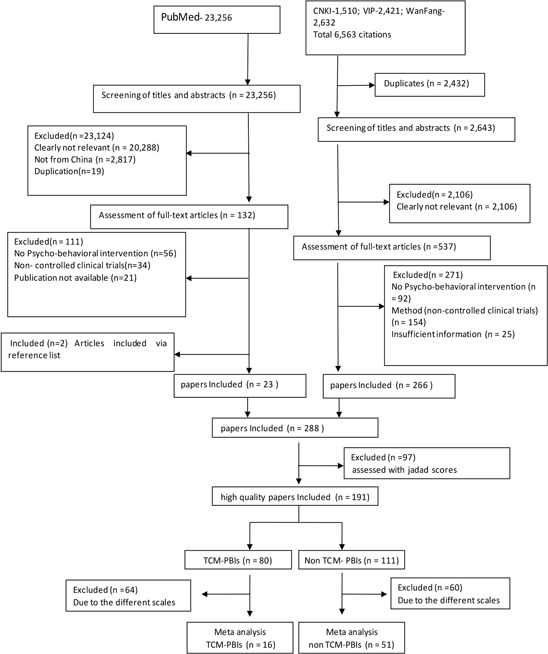
Flow chart illustrating the identification and screening of studies

All case-controlled clinical trials examining the association between PBIs and QoL in Chinese cancer patients published prior to September 01, 2013 were included. Articles were identified by computer-based searches of the following databases: MEDLINE via PubMed, the China National Knowledge Infrastructure (CNKI) database (1911–2013), the Chinese Scientific Journal Database (VIP) (1989–2013) and the Wanfang Database (1994–2013). Searches were conducted by combining the following terms: (“cancer” OR “tumor” OR “neoplasm” OR “oncology”), AND (“acupoint” OR “acupuncture” OR “moxibustion” OR “TCM Five Element” OR “massage” OR “Qigong” OR “Tai Chi” OR “music therapy” OR “dietary therapy” OR “relax” OR “hypnosis” OR “imagery” OR “psychology” OR “psychosis” OR “cognitive therapy” OR “behavior therapy” or “behavior intervention”) AND (“quality of life” OR “effect”) AND (“China”). Fig. 1 summarizes the flow of information that was used for selection of high-quality clinical trials used in the final meta-analysis.

### Inclusion and exclusion criteria

Articles were further refined through a filtering process based upon the following eligibility criteria: (1) Participants: All subjects were adults aged 18 years or older who were formally diagnosed with cancer of any type (solid and hematologic), any tumor stage, any type of treatment mode and any time since diagnosis. Cancer types were sub-grouped into 10 categories according to the International Classification of Diseases-Version 10 (ICD-10). (2) Interventions: PBIs were defined as non-pharmacological approaches and included TCM and non-TCM PBIs. TCM PBIs were defined as only those that included acupuncture, therapeutic Chinese massage, Tai Chi, Qigong, TCM DS and TCM FEMI. In TCM DS, patients were given individualized diet according to the properties of their body, which were based on the Traditional Chinese Medicine Five Elements Theory. While in TCM FEMI, music were developed based on Traditional Chinese Medicine Five Elements Theory, in contrast with other light music. Non-TCMs included relaxation training, cognitive behavioral therapy (CBT), music therapy, guided imagery, psychotherapy and hypnosis, individually or in combination. (3) Controls: No PBI treatment (standard of care) and active (placebo) control conditions were both considered. Studies comparing two or more specific psycho-behavioral interventions without the use of a control condition were excluded. (4) Outcomes: The primary outcome was health-related QoL post-treatment. In this paper, we used QoL as defined by the World Health Organization as “the individuals' perceptions of their position in life in the context of the culture and value systems in which they live and in relation to their goals, expectations, standards and concerns” [13]. The concept of health-related QoL (HR QoL) encompasses those aspects that can be clearly shown to affect health, either physical or mental [14]. In this meta-analysis, the overall QoL in cancer patients was measured by the Karnofsky Performance Score (KPS), the Quality of Life Questionnaire Core-3 (QoL-C30) or the Quality of Life Questionnaire for Chinese Cancer Patients with Chemo-biotherapy (QLQ-CCC). Tools used to evaluate specific physical aspects of QoL included the Visual Analogue Scale (VAS), the Pittsburgh Sleep Quality Index (PSQI) and the Piper Fatigue Scale. Instruments for assessing psychological aspects of QoL included the Self-rating Anxiety Scale (SAS), Self-rating Depression Scale (SDS) and the Profile of Mood States-Short Form (POMS-SF). Secondary outcomes included chemo-radiotherapy-induced side effects, the number of T cells, NK cells and B cells post-surgery side effects and other cancer related symptoms, including pain, fever, fatigue and distress. (5) Studies: Only controlled trials were considered, including randomized controlled trials and 62 non-randomized clinical studies. This refinement procedure yielded 288 articles. The references included in this review can be found in the Appendix (online only).

### Risk of bias assessment

Of the 288 articles reviewed, only high-quality articles were included in the meta-analyses, as determined using the modified Jadad scale [15]. Four components associated with the risk of bias were assessed: sequence generation, blinding, reason for dropping out and allocation concealment. When the RCT includes paper that offer only general comments without a detailed description of randomization and blinding, one point for each index was given. One point was added when there was a detailed and appropriate description. However, one point was deducted when the description was inappropriate. When the specified number and reasons for drop-outs by each subject group were provided, one point was given. When there were no drop-outs, this was specifically stated. If the total score was ≥2 points, the article was considered high quality [15]. Among these papers, 191 were scored greater than 2. Of those, 67 studies employed similar evaluation instruments that were sufficiently homogeneous to be entered into the meta-analysis. These included 51 non-TCM PBIs and 16 TCM PBIs articles (Fig. 1). None of the non-randomized clinical studies were entered into the meta-analysis because their Jadad scores were <2.

### Documentation of study characteristics

All relevant information from the selected high-quality studies was extracted, including clinical sample characteristics, type and duration of PBIs, type of cancer, stage of cancer, type of QoL outcomes and measurement tools. All features were assessed by five independent experts and any discrepancies between reviewers were resolved by discussion.

### Statistical methods

Original data from all of the studies were compiled in Excel software. Statistical analyses were conducted using Review Manager 5.0, which was supplied freely by the Cochrane cooperation net for meta-analysis [16]. As a first step, we calculated the effect size for each study to represent the magnitude of the association between PBIs and QoL. Individual effect sizes were then synthesized to generate an overall effect size using a random or fixed effects model, according to the heterogeneity level, and weighted by the inverse of the variance. We used the effect size Cohen's d or Odds Ratio as the global effect size. Variability among studies in a systematic review may be termed heterogeneity. Heterogeneity was assessed using Cochrane's Q and I^2^, which calculated the proportion of variation attributed to heterogeneity. I^2^ < 25% was considered low, 50% moderate and 75% high. If I^2^ > 70%, potential sources of heterogeneity were identified by sensitivity analyses. This was performed by excluding one study according to study quality and investigating the influence of methodological quality of the combined estimates [17]. Finally, subgroup analysis was performed for categorical variables, comparing the pooled random or fixed effect size with separate estimates of tau (τ) for each subgroup using the Z test [18]. The criterion for significance was set as α = 0.05 for all analyses.

## RESULTS

### Characteristics of articles enrolled in the meta-analysis

We conducted a search for all studies related to PBIs in Chinese cancer patients that have ever been published in the Chinese literature and elsewhere. A total of 67 papers reached the criteria for entrance into the meta-analysis when analyzing the association between PBIs and the QoL (Table 1 and Data Supplemental Table DS2). There were 16 TCM PBIs (Table 1) and 51 non-TCM PBIs studies (Data Supplemental Table DS2) that included 6,806 cancer patients. In the final analysis, there were a total of 9 PBI methods that were fully evaluated: three TCM PBIs (acupuncture, Chinese massage and TCM DS (Data Supplemental Fig. DS1A) and six non-TCM PBIs (relaxation training, cognitive behavioral therapy (CBT), music therapy, guided imagery, psychotherapy and hypnosis (Data Supplemental Fig. DS1)). Unfortunately, high quality trials on TCM FEMI, Qigong and Tai Chi were not analyzed due to failure to meet eligibility criteria for the meta-analysis. Characteristics of the 16 TCM and 51 non-TCM PBIs papers that were included in meta-analysis are presented in Data Supplemental Tables DS1 and DS3.

**Table 1:** Descriptive summary of TCM PBIs clinical studies included in this meta-analysis

variable	Total sample (*n* = 16)		Acupuncture (*n* = 11)		Massage (*n* = 4)		Dietary (*n* = 1)	
	No.	%	No.	%	No.	%	No.	%
**Population**								
Total No. of patients	1649	100.00	968	58.70	595	36.08	86	5.22
Median age, years	55		61		52		54	
Females (%)	68		50		53		100	
**Type of cancer**								
mixed malignant neoplasms	7	37.50	5	31.25	1	6.25	1	6.25
digestive organs	10	62.50	8	50.00	2	12.50	0	0.00
respiratory and intrathoracic organs	6	37.50	5	31.25	1	6.25	0	0.00
lip, oral cavity and pharynx	2	12.50	1	6.25	1	6.25	0	0.00
breast	5	31.25	4	25.00	1	6.25	0	0.00
female genital organs	6	37.50	4	25.00	1	6.25	1	6.25
lymphoid, haematopoietic	3	18.75	2	12.50	1	6.25	0	0.00
bone and articular cartilage	0	0.00	0	0.00	0	0.00	0	0.00
male genital organs	0	0.00	0	0.00	0	0.00	0	0.00
eye, brain and other parts of central nervous system	1	6.25	1	6.25	0	0.00	0	0.00
urinary tract	0	0.00	0	0.00	0	0.00	0	0.00
thyroid and other endocrine glands	0	0.00	0	0.00	0	0.00	0	0.00
**Stage of tumor**								
I-II	0	0.00	0	0.00	0	0.00	0	0.00
III-IV	4	25.00	2	12.50	1	6.25	1	6.25
not reported	10	62.50	7	43.75	3	18.75	0	0.00
both	1	6.25	1	6.25	0	0.00	0	0.00
**Duration, days**								
median, days	27		23		34			
minimum	5		5		5			
maximum	84		56		84			
not reported	3		1		1		1	
**Outcomes**								
QoL score	5	18.75	4	25.00	0	0.00	1	6.25
appetite	1	6.25	1	6.25	0	0.00	0	0.00
sleeplessness	0	0.00	0	0.00	0	0.00	0	0.00
anemia	0	0.00	0	0.00	0	0.00	0	0.00
nausea and vomiting	0	0.00	0	0.00	0	0.00	0	0.00
thirst	0	0.00	0	0.00	0	0.00	0	0.00
abdominal distension	1	6.25	1	6.25	0	0.00	0	0.00
diarrhea	2	12.50	2	12.50	0	0.00	0	0.00
constipation	0	0.00	0	0.00	0	0.00	0	0.00
fatigue	2	12.50	2	12.50	0	0.00	0	0.00
radiation pneumonia	0	0.00	0	0.00	0	0.00	0	0.00
hair loss	0	0.00	0	0.00	0	0.00	0	0.00
hiccup	0	0.00	0	0.00	0	0.00	0	0.00
post-surgery lymph swelling	0	0.00	0	0.00	0	0.00	0	0.00
post-surgery pain	2	12.50	1	6.25	1	6.25	0	0.00
post-surgery insomnia	2	12.50	1	6.25	1	6.25	0	0.00
number of T cells, NK cells and B cells	0	0.00	0	0.00	0	0.00	0	0.00
distress	3	18.75	3	18.75	0	0.00	0	0.00
intestinal function	4	25.00	2	12.50	2	12.50	0	0.00
pain induced by tumor	2	12.50	1	6.25	1	6.25	0	0.00
**Evaluation tools**								
KPS	4	25.00	3	18.75	0	0.00	1	6.25
QLQ-C30	0	0.00	0	0.00	0	0.00	0	0.00
QOL score without specific information	1	6.25	1	6.25	0	0.00	0	0.00
SCL90	0	0.00	0	0.00	0	0.00	0	0.00
SDS	4	25.00	3	18.75	1	6.25	0	0.00
SAS	2	12.50	1	6.67	1	6.67	0	0.00
HAMA	1	6.25	1	6.25	0	0.00	0	0.00
HAMD	3	18.75	3	18.75	0	0.00	0	0.00
VAS	2	12.50	1	6.25	1	6.25	0	0.00
PSQI	2	12.50	1	6.25	1	6.25	0	0.00
Piper.PFS	2	12.50	2	12.50	0	0.00	0	0.00
other tools	8	50.00	4	25.00	3	18.75	1	6.25
**Publication types**								
journal articles	16	100.00	11	68.75	4	25.00	1	6.25
conference proceedings	0	0.00	0	0.00	0	0.00	0	0.00
dissertations	0	0.00	0	0.00	0	0.00	0	0.00
publication year								
2013	0	0.00	0	0.00	0	0.00	0	0.00
2012	6	37.50	4	25.00	1	6.25	1	6.25
2011	2	12.50	1	6.25	1	6.25	0	0.00
2010	2	12.50	2	12.50	0	0.00	0	0.00
2009	2	12.50	1	6.25	1	6.25	0	0.00
2008	2	12.50	1	6.25	1	6.25	0	0.00
2007	0	0.00	0	0.00	0	0.00	0	0.00
2006	1	6.25	1	6.25	0	0.00	0	0.00
2005	1	6.25	1	6.25	0	0.00	0	0.00
2004	0	0.00	0	0.00	0	0.00	0	0.00
2003	0	0.00	0	0.00	0	0.00	0	0.00
2002	0	0.00	0	0.00	0	0.00	0	0.00
2001	0	0.00	0	0.00	0	0.00	0	0.00
2000	0	0.00	0	0.00	0	0.00	0	0.00
Total No. of publications	16	100.00	11	68.75	4	25.00	1	6.25

Cancer types were sub-grouped into 10 categories according to the ICD-10. Ranked by the total numbers of studies, the three most prevalent types of cancer for the TCM PBIs papers were malignant neoplasms of the digestive organs (26%), female genital organs (15%) and respiratory and intrathoracic organs (15%) (Data Supplemental Fig. DS1B). For the non-TCM PBIs articles, the three most frequently reported types of cancer were malignant neoplasms of the breast (24%), digestive organs (15%) and female genital organs (14%) (Data Supplemental Fig. DS1C).

### PBIs for cancer patients

**Figure 2:**
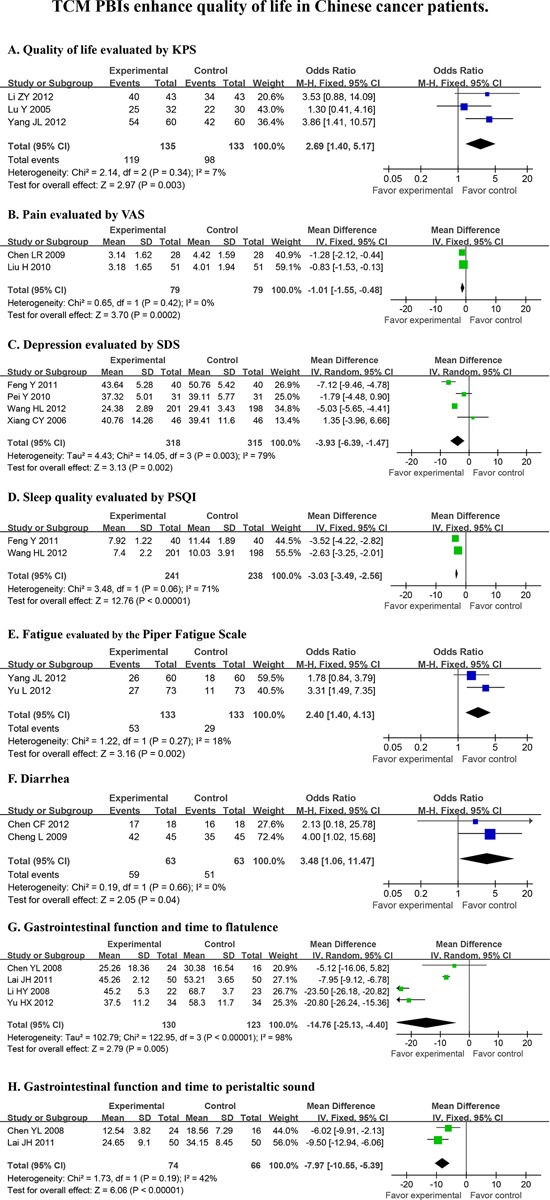
TCM PBIs enhance quality of life in cancer patients Random effects were used for statistical evaluation of all PBIs that were tested for overall quality of life (KPS^a^), pain (VAS^b^), depression (SDS^c^), sleep (PSQI^d^), fatigue and gastrointestinal function. KPS^a^ (Karnofsky Performance Score); VAS^b^ (Visual analogue scale); SDS^c^ (Self-rating depression scale); PSQI^d^ (Pittsburgh sleep quality index).

The meta-analysis confirmed that TCM PBIs enhanced global QoL for Chinese cancer patients (OR = 2.69 *P* = 0.003, Fig. 2A). With respect to individual symptoms, TCM PBIs relieved pain (*d* = −1.01 *P* = 0.0002, Fig. 2B), reduced the level of depression (*d* = − 3.93 *P* = 0.002, Fig. 2C), improved sleep quality (*d* = − 3.03 *P* < 0.00001, Fig. 2D), alleviated fatigue (OR = 2.40 *P* = 0.002, Fig. 2E), eased diarrhea (OR = 3.48 *P* = 0.04, Fig. 2F) and promoted intestinal function after surgery (*d* = − 14.76 *P* = 0.005, Fig. 2G and *d* = − 7.97 *P* < 0.00001, 2H).

Compared with TCM PBIs, our meta-analysis findings demonstrated that non-TCM PBIs also reduced functional impairments in cancer patients (*d* = 15.35 *P* < 0.00001, Data Supplemental Fig. DS2A) and increased overall QoL (*d* = 11.18 *P* < 0.00001, Data Supplemental Fig DS2B and *d* = 12.70 *P* < 0.00001, Data Supplemental Fig DS2C). The non-TCMs reduced depressive symptoms (d = − 10.27 *P* < 0.00001, Data Supplemental Fig DS2D) and anxiety (*d* = − 10.61 *P* < 0.00001, Data Supplemental Fig DS2E), improved global mood (*d* = −14.69 *P* < 0.00001, Data Supplemental Fig DS2F), relieved pain (*d* = − 1.45 *P* = 0.002, Data Supplemental Fig DS2G) and improved sleep quality (*d* = − 5.91 *P* < 0.00001, Data Supplemental Fig. DS2H). It also shortened the time to first flatulence (*d* = − 4.81 *P* = 0.0002, Data Supplemental Fig. DS2I) in cancer patients. However, no significant effects were found for non-TCM PBIs on fatigue or gastrointestinal symptoms.

### TCM PBIs improve QoL

#### Acupuncture

**Figure 3:**
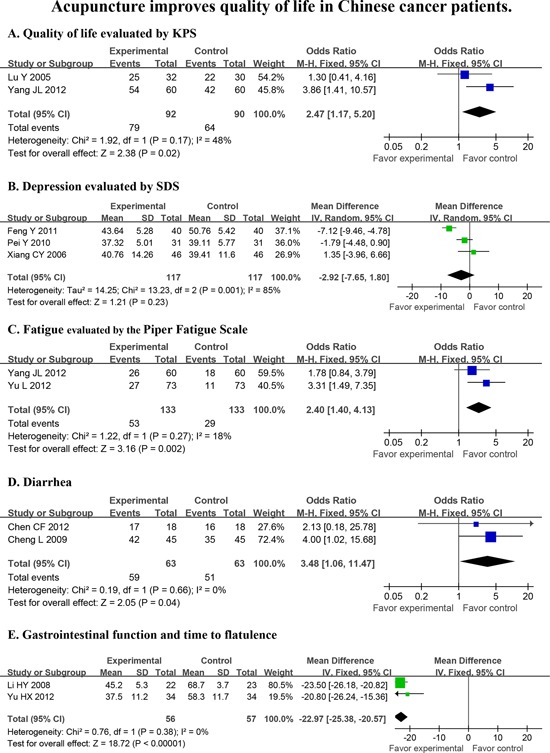
Acupuncture improves quality of life in Chinese cancer patients Quality of life (KPS), depression (SDS), fatigue, diarrhea and time to flatulence were evaluated in a statistical model that used random effects.

Eleven high quality clinical trials involving 968 cancer patients evaluated acupuncture and entered the meta-analysis. These results showed that acupuncture improved overall QoL in cancer patients as assessed by the KPS remission rate scale (OR = 2.47 *P* = 0.02, Fig. 3A). In particular, acupuncture relieved fatigue (OR = 2.40 *P* = 0.002, Fig. 3C), reduced diarrhea (OR = 3.48 *P* = 0.04, Fig. 3D) and shortened the time to first flatulence (*d* = − 22.97 *P* < 0.00001, Fig. 3E) despite failing to relieve symptoms of depression (*P* = 0.23, Fig. 3B). A majority of these trials (8 out of 11) were conducted in patients with malignant neoplasms of the digestive system.

#### Therapeutic chinese massage

**Figure 4:**
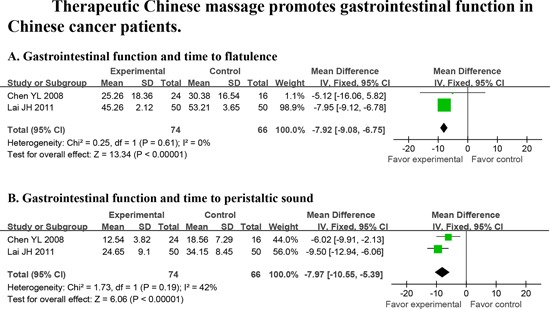
Massage promotes gastrointestinal function in cancer patients Random effects were used to analyze time to first flatulence and intestinal peristaltic sound.

The effect of Chinese massage on QoL was assessed in 4 trials involving 595 patients. Chinese massage reduced time to first flatulence (*d* = − 7.92, *P* < 0.00001, Fig. 4A) and intestinal peristaltic sound following surgery (*d* = − 7.97, *P* < 0.00001, Fig. 4B).

#### TCM dietary supplement

One article on TCM DS was entered into the meta-analysis [19]. In this trial, 43 advanced ovarian cancer patients were assigned to receive chemo-therapy plus two years of TCM DS and compared to patients given only chemo-therapy. Quality of life was significantly improved in patients who underwent TCM DS compared with the pure chemotherapy group. The former group also displayed a significant increase in CD4+ lymphocytes and NK activity with a reduction in the number of CD8+ cells [19].

#### Tai chi

No evaluation of Tai Chi was performed in this meta-analysis because of excess heterogeneity in the scales used to evaluate QoL. However, five papers of high quality reported a positive association between Tai Chi and QoL in cancer patients [20–24]. Indeed, in one study that scored 4 according to the Jadad Scale, 67 patients were assigned to receive a 6-month intervention of Tai Chi as compared to a control group (*n* = 71). Patients who underwent Tai Chi had less post-operative upper limb lymph-edema [24]. Another 4 papers with a Jadad score of 2 reported that Tai Chi increased circulating concentrations of IgA, IgG and IgM [23] and improved lung function [21], sleep quality [20, 25] and limb muscle strength in cancer patients [21]. Tai Chi also alleviated fatigue [20] and reduced distress [23].

#### TCM five element musical intervention

TCM FEMI was assessed in 3 clinical trials that were excluded from the meta-analysis because Jadad scores of the studies were <2. In one study, 40 cancer patients were assigned to TCM FEMI at the beginning of chemotherapy. TCM FEMI was practiced once a day for three cycles (4 weeks) that were synchronic with chemotherapy. Compared to routine chemotherapy alone, TCM FEMI reduced cancer-related fatigue as assessed by the Brief Fatigue Inventory [26]. Another 2 trials involving 143 patients reported that TCM FEMI reduced symptoms of depression and improved global QoL in Chinese cancer patients [27, 28].

#### Qigong

Although there were an insufficient number of clinical trials with Qigong and cancer patients that were identified in the original screen, one was of high enough quality to be included in the meta-analysis [29]. This clinical trial showed that the practice of Qigong in women with breast cancer resulted in less fatigue and improved QoL (*n* = 49) as compared to the control group (*n* = 47). However, no significant differences were observed for sleep quality.

## DISCUSSION

In this report, we searched for all PBIs that were used in clinical cancer trials in China that were published prior to September 1, 2013, regardless of whether the paper was published in Chinese or English. There were 67 papers that met our pre-defined inclusion criteria, amounting to 6,806 cancer patients. We were surprised by the limited number of papers on the use of TCM PBIs for Chinese cancer patients that qualified for entry into this meta-analysis, particularly since TCMs originated and are mostly practiced in China. Despite the growing acceptance of a variety of PBIs around the world, there has been no comprehensive evaluation of high-quality, well-controlled clinical studies on the use of TCM PBIs, even in China. Many individual TCM trials have reported conflicting results. This formal analysis thereby serves as the first complete and comprehensive analysis of all TCM-PBIs on QoL in cancer patients in China. This meta-analysis demonstrates that TCM PBIs (Fig. 2), particularly acupuncture (Fig. 3) and therapeutic Chinese massage (Fig. 4), exert a positive and beneficial impact on QoL in Chinese cancer patients.

A total of 51 non-TCM PBIs papers and 16 TCM PBIs studies qualified for entry into the meta-analysis when analyzing the association between PBIs and QoL (Table 1 and Data Supplemental Table DS2). A number of QoL measures, including pain, depression, insomnia, fatigue, diarrhea and intestinal dysfunction were relieved by TCM PBIs (Table 1 and Data supplemental Table DS2). Acupuncture, the most frequently applied form of TCM PBIs, improved QoL (Fig. 3A) by relieving depression, fatigue, diarrhea and gastrointestinal distress (Fig. 3B–3E). Therapeutic Chinese massage reduced time to first flatulence (Fig. 4A) and intestinal peristaltic sound (Fig. 4B). This reduction in symptoms is important for reducing the risk of paralytic ileus by stimulating digestive propulsion, particularly following gastrointestinal surgery. On the other hand, non-TCM PBIs increased overall QoL (Data Supplemental Fig. DS2A–2C) by relieving depression (Data Supplemental Fig. DS2D), anxiety (Data Supplemental Fig. DS2E), global negative mood (Data Supplemental Fig. DS2F), pain (Data Supplemental Fig. DS2G), sleep loss (Data Supplemental Fig. DS2H) and poor gastrointestinal function following surgery (Data Supplemental Fig. DS2I). Comparing TCM and non-TCM PBIs, this meta-analysis clearly establishes that both are of significant benefit to the QoL in Chinese cancer patients. Importantly, TCM PBIs specifically relieved fatigue, eased diarrhea and improved gastrointestinal function followed surgery (Fig. 2E–2H).

Several individual clinical studies have consistently established that acupuncture relieves fatigue and reduces diarrhea [30–32]. Our meta-analysis with a much larger cohort of patients confirms these beneficial effects of acupuncture on fatigue and diarrhea. It is important to note that acupuncture did not improve symptoms of depression, whereas a significant improvement in depressive symptoms has been reported in previous studies [33–35]. This discrepancy may be at least partially caused by the heterogeneity of outcomes reported among these papers and also because there are likely to be different symptoms of depression in different types of cancers. On the other hand, other studies have also shown that massage decreases severity of gastrointestinal symptoms [36–38]. Together with our finding that therapeutic Chinese massage reduces time to first flatulence and peristaltic sound following surgery, this form of TCM can be confidently viewed as a beneficial treatment for improving gastrointestinal function. Unfortunately, very few trials using Tai Chi as a cancer intervention were available to be included in our meta-analysis, so there is currently insufficient evidence to clarify the effectiveness of Tai Chi on QoL in Chinese cancer patients. However, other clinical studies have reported that Tai Chi improves both mental and physical aspects of QoL, including distress, fatigue, sleep quality and limb, lung and immune functions [39–41].

Although our study provided insufficient evidence to reach conclusions for TCM FEMI, Qigong and Tai chi, the three methods indeed have a medical basis for improving QoL in cancer patients. Previous meta-analytic reviews suggested that Qigong and Tai chi improve cancer-specific QoL [42], fatigue, immune function and cortisol level of cancer patients [43]. Qigong decreased the rate of leukopenia in breast cancer patients following chemotherapy [44]. Furthermore, Qigong selectively inhibited phosphorylation levels of Akt and extracellular signal-regulated kinase pathways in cancer cells but not in normal cellular survival pathways [45]. Similarly, Tai chi increased IL-6 and decreased IL-2 levels which was positively related to an improvement in QoL in breast cancer patients [46]. Although few studies supported TCM FEMI improving QoL in cancer patients, this practice of using for musical composition based on the five elements of TCM (Earth, Metal, Water, Wood, and Fire) created a relaxed state for patients [47]. Consistent with our study, Qigong, Tai chi and TCM FEMI are also promising interventions in improving QoL in cancer patients, but more research is needed to support this preliminary conclusion.

Previous meta-analyses have consistently established that subsets of non-TCM improve QoL in cancer patients [48–50]. However, potential QoL benefits have not been assessed using a large scale meta-analysis in Chinese cancer patients treated with TCM PBIs. Even though the focus of our analysis was on QoL in patients treated with TCM PBIs, we also sought to confirm the positive effects of aggregate non-TCMs on QoL in Chinese patients. In addition to TCM and non-TCM PBIs, there are a number of individualized treatment approaches that span the full spectrum of cancer care to prevent cancer-related symptoms and post-treatment side effects [51], including fatigue, pain, diarrhea, depression and gastrointestinal dysfunction [52, 53]. Due to concerns that many anesthetics reduce gastrointestinal mobility, as well as inducing other side effects [54, 55], non-pharmacological analgesia such as structured attention and self-hypnotic relaxation has been shown reduce both anxiety and pain [56].

The TCM PBIs evaluated in this meta-analysis may have promising effects following anesthesia by reducing the incidence or severity of post-operative ileus. Chemotherapy for cancer patients, such as can occur with the use of fluorouracil (5-FU), often leads to severe diarrhea [57, 58]. Similarly, post-surgery gastrointestinal dysfunction frequently occurs following colorectal cancer surgery [59, 60]. Breast cancer patients often suffer from poor QoL [61], with more than half of them experiencing fatigue [62–65], the most frequently reported symptom [66]. Unfortunately, limited treatments are available for effectively managing these symptoms in cancer patients [67, 68]. Recent evidence suggests that TCM can be an effective individualized strategy for cancer patients [69]. Coupled with the results of this meta-analysis, it seems quite reasonable to conclude that TCM PBIs are effective interventions that should be used in personalized medicine for treating patient-specific symptoms.

Low QoL is strongly related to poor prognosis and low survival rate of cancer patients [70–72]. This is the first work with Chinese cancer patients to definitively demonstrate that TCM PBIs significantly improve their QoL. This occurs because TCM is associated with relieving pain, depression, sleep loss, fatigue, diarrhea and gastrointestinal dysfunction. TCM PBIs represent beneficial adjunctive therapy for cancer patients.

## SUPPLEMENTARY FIGURES AND TABLES


