# Supplementary material for: Practice of traditional Chinese medicine for psycho-behavioral intervention improves quality of life in cancer patients: A systematic review and meta-analysis
Source: Oncotarget. 2015 Oct 15;6(37):39725–39. doi: 10.18632/oncotarget.5388 (PMC4741858; doi:10.18632/oncotarget.5388)
Supplement: Supplementary file 1 [file oncotarget-06-39725-s001.pdf]

SUPPLEMENTARY FIGURES AND TABLES

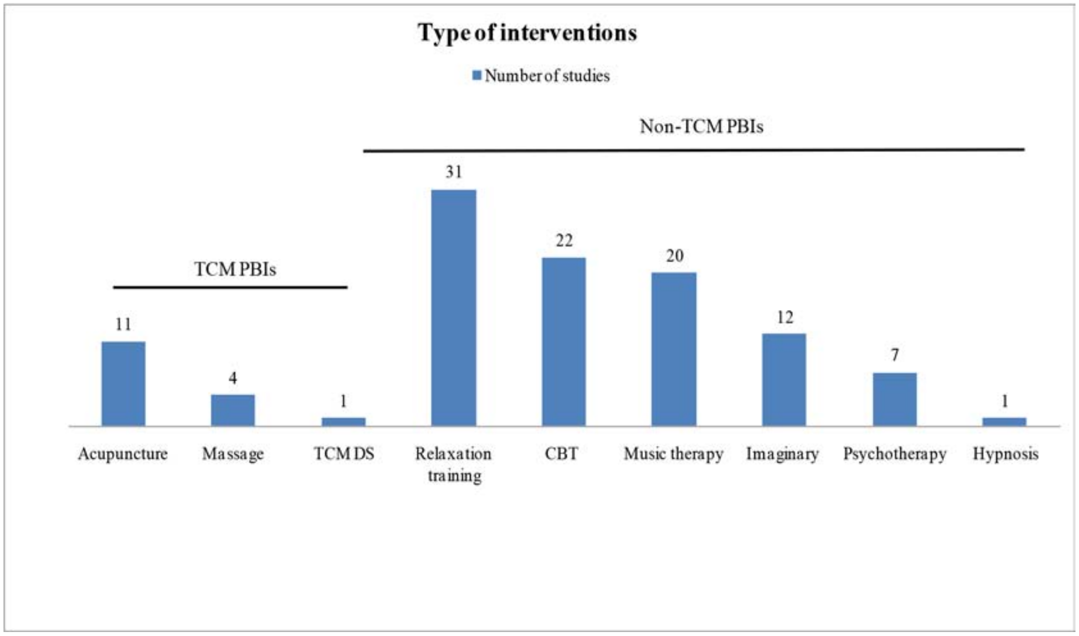

Supplementary Figure S1: Categories of TCM and non-TCM clinical studies of cancer patients in the high-quality data base used for this meta-analysis. A. The components of interventions. Sixteen articles evaluated TCM PBIs, and 51 papers reported the effects of non-TCM PBIs. Among non-TCM PBI clinical trials, twenty-seven reported more than 2 types of PBIs.

(*continued*)

B. The components of cancer types in TCM PBIs studies.

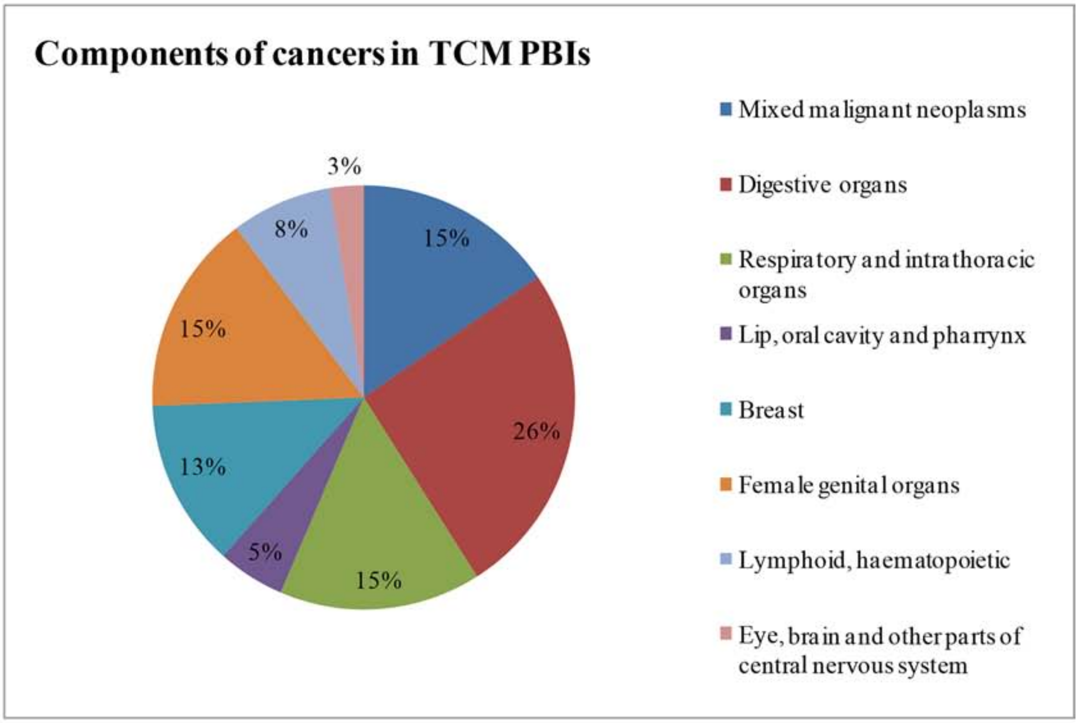

C. The components of cancer types in non-TCM PBIs studies.

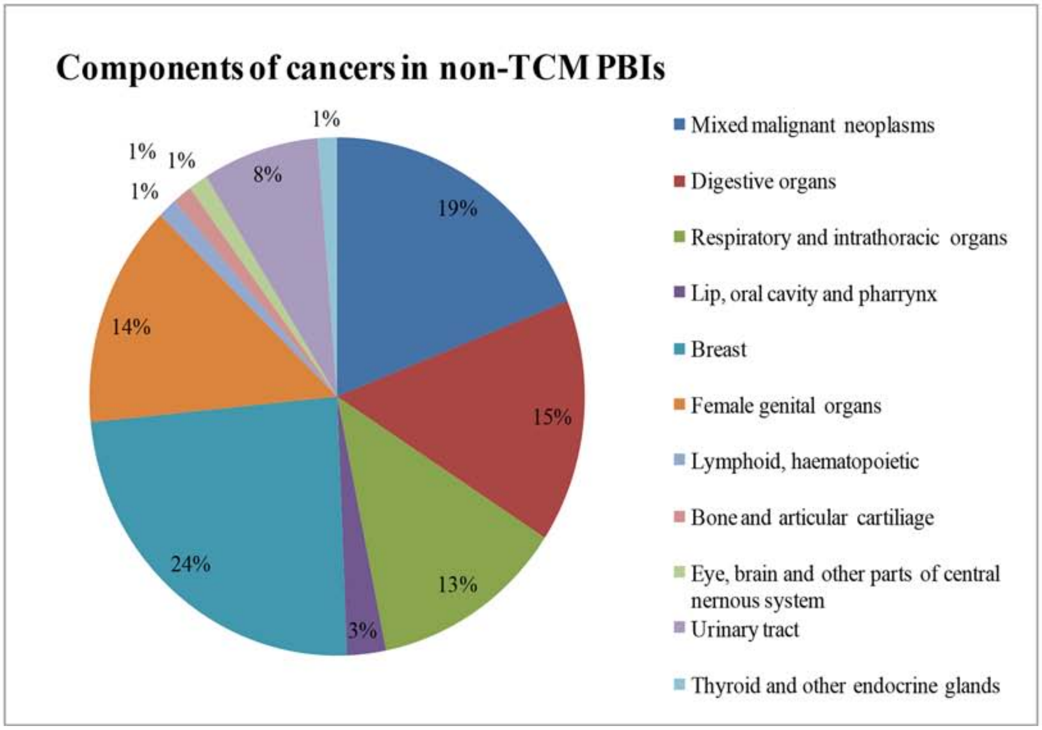

Supplementary Figure S1: (*continued*) B. Types of cancer reported in the TCM PBI studies. **C.** Types of cancer in subjects in the non-TCM PBI studies.

(*continued*)

A. Quality of life evaluated by KPS

Study or Subgroup	Experimental Mean	Experimental SD	Experimental Total	Control Mean	Control SD	Control Total	Weight	Mean Difference IV, Fixed, 95% CI
Cao DL 2006	75.5	5.7	45	60.2	5.1	45	19.3%	15.30 [13.07, 17.53]
Xie Z 2001	75.6	5.8	124	60.2	5.2	136	53.4%	15.40 [14.06, 16.74]
Xie Z 2006	75.5	5.7	45	60.2	5.1	130	27.2%	15.30 [13.42, 17.18]
**Total (95% CI)**			**214**			**311**	**100.0%**	**15.35 [14.37, 16.34]**

Heterogeneity: Chi² = 0.01, df = 2 (P = 1.00); I² = 0%
Test for overall effect: Z = 30.63 (P < 0.00001)

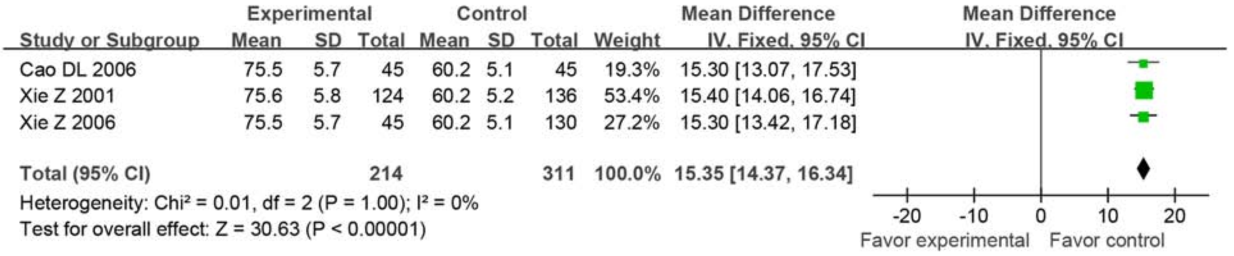

B. Quality of life evaluated by QLQ-C30

Study or Subgroup	Experimental Mean	Experimental SD	Experimental Total	Control Mean	Control SD	Control Total	Weight	Mean Difference IV, Random, 95% CI
Bai SM 2004	61.15	26.57	24	52.46	21.71	21	4.5%	8.69 [-5.42, 22.80]
Chen JJ 2011	63.59	19.42	33	49.11	21.37	33	5.9%	14.48 [4.63, 24.33]
Cheng K 2006	27.03	4.6	15	35.47	5.88	15	8.0%	-8.44 [-12.22, -4.66]
Guo Z 2013	58.8	12.2	89	51.12	12.82	89	8.0%	7.68 [4.00, 11.36]
Le GA 2005	47.92	14.2	32	34.64	16.5	32	6.8%	13.28 [5.74, 20.82]
Li CM 2012	67.19	26.15	55	60.25	23.12	55	6.2%	6.94 [-2.28, 16.16]
Tian JM 2010	48	13	48	35	15	48	7.4%	13.00 [7.38, 18.62]
Tian XY 2012	65.41	10.03	40	54.28	9.47	40	7.8%	11.13 [6.86, 15.40]
Wang JP 2006	61.51	22.02	204	45.72	22.07	204	7.8%	15.79 [11.51, 20.07]
Wang WL 2013	65.11	9.12	106	50.39	11.2	100	8.2%	14.72 [11.92, 17.52]
Xu L 2012	54.53	15.34	66	34.34	17.65	33	6.9%	20.19 [13.12, 27.26]
Yao YY 2009	62.5	10.29	40	52.5	15.7	40	7.4%	10.00 [4.18, 15.82]
Ye LX 2013	66	10	65	49	10	60	8.0%	17.00 [13.49, 20.51]
Zhang H 2009	70.47	11.35	46	57.97	20.1	46	7.1%	12.50 [5.83, 19.17]
**Total (95% CI)**			**863**			**816**	**100.0%**	**11.18 [6.79, 15.58]**

Heterogeneity: Tau² = 59.47; Chi² = 136.57, df = 13 (P < 0.00001); I² = 90%
Test for overall effect: Z = 4.99 (P < 0.00001)

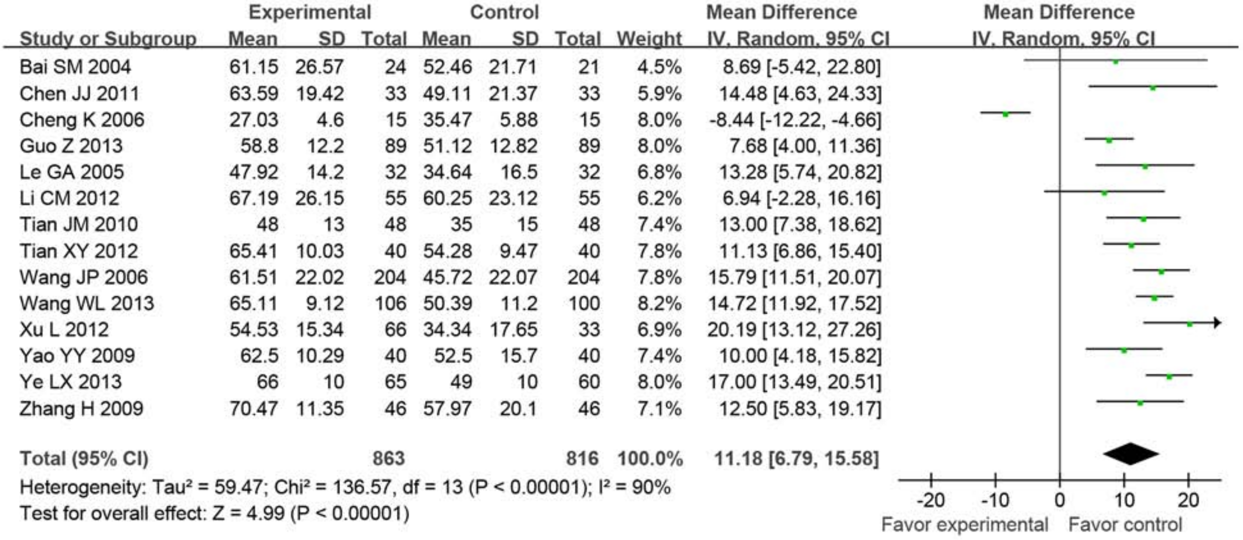

C. Quality of life evaluated by QLQ-CCC

Study or Subgroup	Experimental Mean	Experimental SD	Experimental Total	Control Mean	Control SD	Control Total	Weight	Mean Difference IV, Fixed, 95% CI
Cao DL 2006	47.1	3.5	45	34.4	3.4	45	15.0%	12.70 [11.27, 14.13]
Xie Z 2001	47.2	3.4	124	34.5	3.5	136	43.4%	12.70 [11.86, 13.54]
Xie Z 2006	47.1	3.5	120	34.4	3.4	130	41.6%	12.70 [11.84, 13.56]
**Total (95% CI)**			**289**			**311**	**100.0%**	**12.70 [12.15, 13.25]**

Heterogeneity: Chi² = 0.00, df = 2 (P = 1.00); I² = 0%
Test for overall effect: Z = 45.05 (P < 0.00001)

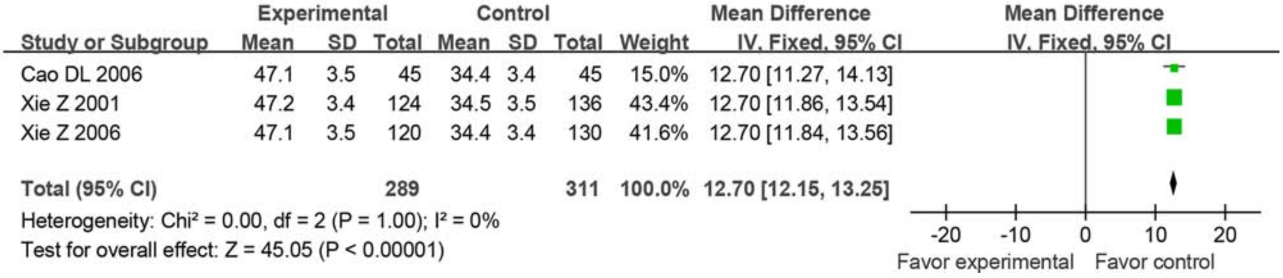

(continued)

D. Depression evaluated by SDS

Study or Subgroup	Experimental Mean	Experimental SD	Experimental Total	Control Mean	Control SD	Control Total	Weight	Mean Difference IV, Random, 95% CI
Cao GR 2007	45.74	8.61	42	50.75	9.02	40	4.8%	-5.01 [-8.83, -1.19]
Chen JZ 2011	46.58	9.83	40	59.78	10.72	40	4.4%	-13.20 [-17.71, -8.69]
Cui JF 2007	34.5	7.3	30	54.8	10.2	30	4.4%	-20.30 [-24.79, -15.81]
Guo Z 2013	51.48	7.54	89	59.05	9.41	89	5.4%	-7.57 [-10.08, -5.06]
Hao YJ 2011	46.63	8.68	30	51.58	6.05	30	4.8%	-4.95 [-8.74, -1.16]
Li HM 2007	40.71	3.56	51	50.18	1.51	50	5.8%	-9.47 [-10.53, -8.41]
Li L 2011	51.23	4.14	40	60.79	3.22	40	5.7%	-9.56 [-11.19, -7.93]
Li XL 2012	44.81	4.37	48	55.06	2.45	48	5.7%	-10.25 [-11.67, -8.83]
Li Y 2012	44.13	8.12	21	49.87	8.61	21	4.2%	-5.74 [-10.80, -0.68]
Lin XY 2010	39.27	8.69	47	45.27	10.74	50	4.7%	-6.00 [-9.88, -2.12]
Liu AM 2006	37.68	4.89	58	49.01	8.56	53	5.3%	-11.33 [-13.96, -8.70]
Lu FM 2013	41.89	8.97	65	51.95	10.02	65	5.0%	-10.06 [-13.33, -6.79]
Mei JH 2011	40.11	6.21	39	50.14	7.42	39	5.1%	-10.03 [-13.07, -6.99]
Peng HZ 2012	43.87	8.86	26	52.41	9.83	26	4.1%	-8.54 [-13.63, -3.45]
Wang WL 2013	40.1	9.2	106	59.12	10.2	100	5.3%	-19.02 [-21.68, -16.36]
Wang YE 2012	44.21	10.42	40	55.35	8.94	40	4.6%	-11.14 [-15.39, -6.89]
Ye LX 2013	41	11	65	51	13	60	4.6%	-10.00 [-14.24, -5.76]
Zhang MF 2008	37.8	6.8	60	59.9	7.3	60	5.4%	-22.10 [-24.62, -19.58]
Zhang WZ 2010	76.43	8.46	58	80.05	9.53	49	4.9%	-3.62 [-7.06, -0.18]
Zhou KN 2010	25.41	2.65	60	32.12	3.79	60	5.8%	-6.71 [-7.88, -5.54]
**Total (95% CI)**			**1015**			**990**	**100.0%**	**-10.27 [-12.16, -8.38]**

Heterogeneity: Tau² = 15.73; Chi² = 224.14, df = 19 (P < 0.00001); I² = 92%
Test for overall effect: Z = 10.64 (P < 0.00001)

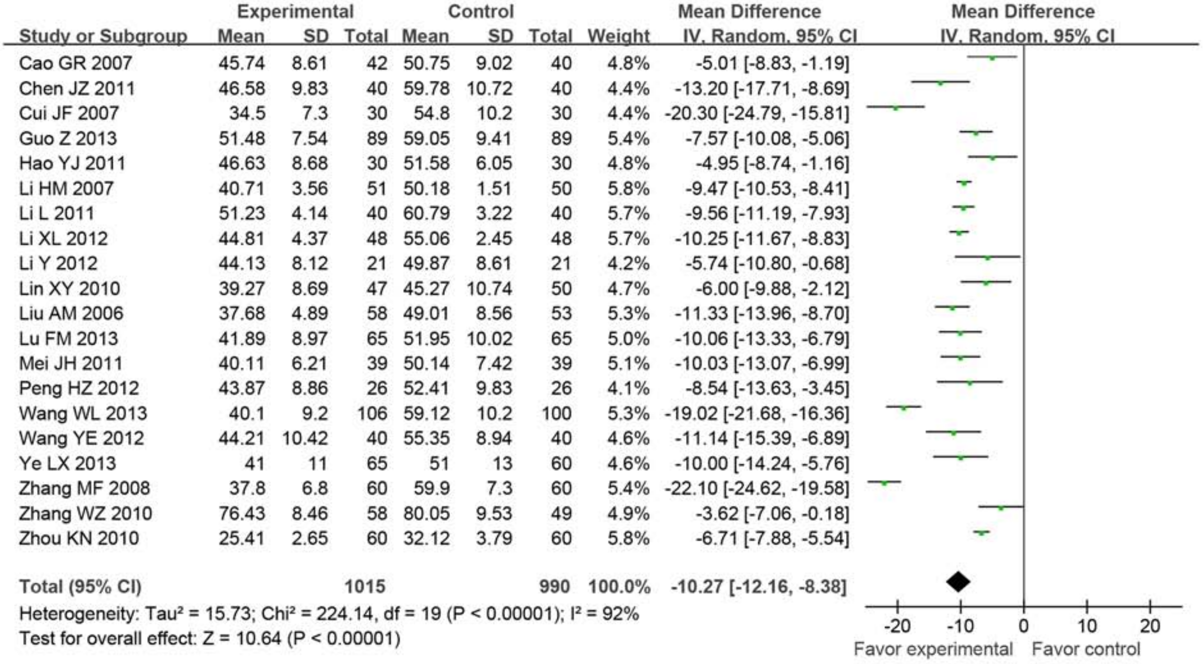

E. Anxiety evaluated by SAS

Study or Subgroup	Experimental Mean	Experimental SD	Experimental Total	Control Mean	Control SD	Control Total	Weight	Mean Difference IV, Random, 95% CI
Bi HW 2011	39.7	3.48	30	64.7	2.7	30	3.8%	-25.00 [-26.58, -23.42]
Cao GR 2007	46.38	8.46	42	50.16	6.52	40	3.7%	-3.78 [-7.04, -0.52]
Cui JF 2007	32.2	5.4	30	46.4	9.3	30	3.7%	-14.20 [-18.05, -10.35]
Guo Z 2013	48.78	8.95	89	55.69	10.01	89	3.8%	-6.91 [-9.70, -4.12]
Hao YJ 2011	47.3	10.45	30	51.95	7.63	30	3.6%	-4.65 [-9.28, -0.02]
Jia HZ 2011	36.03	8.5	35	38.23	7.49	35	3.7%	-2.20 [-5.95, 1.55]
Jiang JF 2008	45.42	6.47	52	62.21	9.2	52	3.7%	-16.79 [-19.85, -13.73]
Li HM 2007	37.82	2.46	51	58.9	1.76	50	3.9%	-21.08 [-21.91, -20.25]
Li L 2011	47.11	5.55	40	59.28	6.11	40	3.8%	-12.17 [-14.73, -9.61]
Li XL 2012	40.79	5.05	48	51.79	2.05	48	3.8%	-11.00 [-12.54, -9.46]
Li Y 2012	45.31	9.67	21	51.63	9.13	21	3.4%	-6.32 [-12.01, -0.63]
Lin XY 2010	35.43	8.76	47	41.62	9.81	50	3.7%	-6.19 [-9.89, -2.49]
Long XL 2011	41.84	9.67	101	48.78	12.13	101	3.7%	-6.94 [-9.97, -3.91]
Lu FM 2013	42.67	7.98	65	52.83	9.86	65	3.7%	-10.16 [-13.24, -7.08]
Mei JH 2011	38.27	6.72	39	49.62	10.03	39	3.7%	-11.35 [-15.14, -7.56]
Mu SY 2010	36.04	8.51	40	38.12	7.38	40	3.7%	-2.08 [-5.57, 1.41]
Niu X 2013	42.44	10.13	35	62.01	12.84	35	3.5%	-19.57 [-24.99, -14.15]
Peng HZ 2012	43.11	9.59	26	50.91	10.01	26	3.5%	-7.80 [-13.13, -2.47]
Wang J 2009	38.23	6.65	33	42.38	6.82	27	3.7%	-4.15 [-7.58, -0.72]
Wang YE 2012	41.31	7.85	40	59.67	8.03	40	3.7%	-18.36 [-21.84, -14.88]
Wen SM 2009	44.23	7.53	62	48.36	6.77	51	3.8%	-4.13 [-6.77, -1.49]
Xiong FZ 2009	40.93	5.23	40	46.18	5.75	40	3.8%	-5.25 [-7.66, -2.84]
Yang XH 2008	35.36	5.79	40	56.18	7.62	40	3.7%	-20.82 [-23.79, -17.85]
Ye LX 2013	40	9	65	51	10	60	3.7%	-11.00 [-14.35, -7.65]
Zhang MF 2008	34.4	6.5	60	55.7	8.4	60	3.8%	-21.30 [-23.99, -18.61]
Zhang WZ 2010	39.96	5.65	58	49.96	5.66	49	3.8%	-10.00 [-12.15, -7.85]
Zhao PT 2008	39.96	5.65	49	42.3	5.23	46	3.8%	-2.34 [-4.53, -0.15]
**Total (95% CI)**			**1268**			**1234**	**100.0%**	**-10.61 [-13.66, -7.56]**

Heterogeneity: Tau² = 62.49; Chi² = 1020.86, df = 26 (P < 0.00001); I² = 97%
Test for overall effect: Z = 6.82 (P < 0.00001)

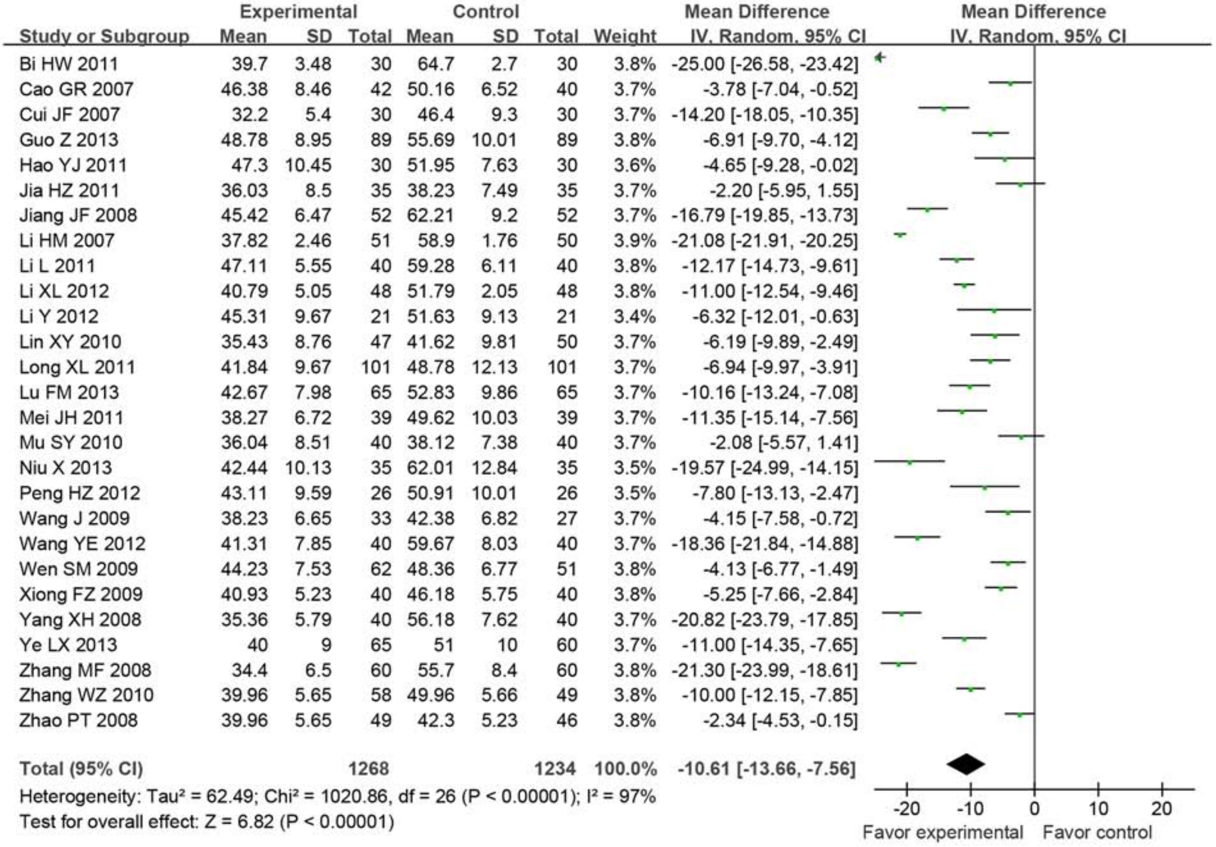

(continued)

F. Global mood evaluated by POMS-SF

Study or Subgroup	Experimental Mean	Experimental SD	Experimental Total	Control Mean	Control SD	Control Total	Weight	Mean Difference IV, Fixed, 95% CI
Wang JP 2006	28.61	12.8	204	46.37	23.27	204	58.5%	-17.76 [-21.40, -14.12]
Zhang H 2009	25.54	6.85	46	35.91	13.32	46	41.5%	-10.37 [-14.70, -6.04]
Total (95% CI)			250			250	100.0%	-14.69 [-17.48, -11.91]

Heterogeneity: Chi² = 6.55, df = 1 (P = 0.01); I² = 85%
Test for overall effect: Z = 10.33 (P < 0.00001)

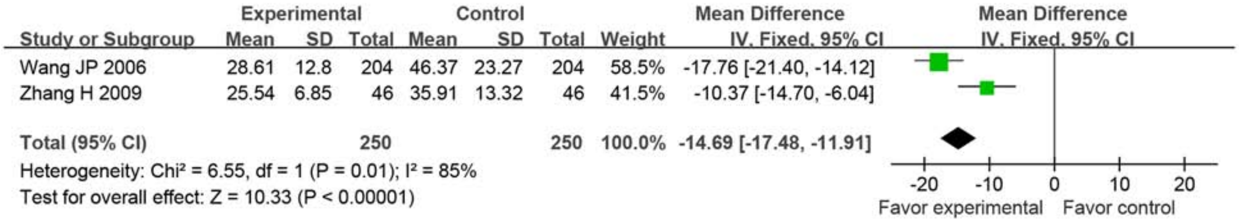

G. Pain evaluated by VAS

Study or Subgroup	Experimental Mean	Experimental SD	Experimental Total	Control Mean	Control SD	Control Total	Weight	Mean Difference IV, Random, 95% CI
Cao HQ 2010	1.87	0.52	15	4.67	0.21	15	20.6%	-2.80 [-3.08, -2.52]
Chen XF 2008	2.67	0.67	26	4.31	1.03	26	19.9%	-1.64 [-2.11, -1.17]
Jia HZ 2011	2.26	1.16	35	3.28	1.36	35	19.3%	-1.02 [-1.61, -0.43]
Li XM 2011	0.98	0.62	60	1.82	0.83	60	20.7%	-0.84 [-1.10, -0.58]
Mu SY 2010	2.27	1.27	40	3.17	1.24	40	19.5%	-0.90 [-1.45, -0.35]
Total (95% CI)			176			176	100.0%	-1.45 [-2.36, -0.53]

Heterogeneity: Tau² = 1.04; Chi² = 110.90, df = 4 (P < 0.00001); I² = 96%
Test for overall effect: Z = 3.10 (P = 0.002)

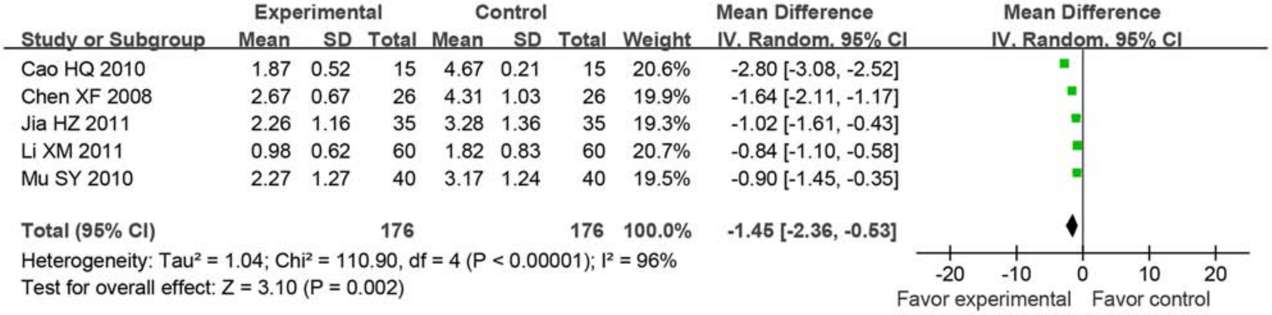

H. Sleep quality and PSQI

Study or Subgroup	Experimental Mean	Experimental SD	Experimental Total	Control Mean	Control SD	Control Total	Weight	Mean Difference IV, Fixed, 95% CI
Ao B 2007	9.57	3.47	21	15.95	2.46	22	14.4%	-6.38 [-8.19, -4.57]
Cao HQ 2010	11.47	1.6	15	17.2	1.01	15	51.1%	-5.73 [-6.69, -4.77]
Xu MH 2010	9.39	3.26	49	15.38	2.59	49	34.5%	-5.99 [-7.16, -4.82]
Total (95% CI)			85			86	100.0%	-5.91 [-6.60, -5.23]

Heterogeneity: Chi² = 0.41, df = 2 (P = 0.81); I² = 0%
Test for overall effect: Z = 16.93 (P < 0.00001)

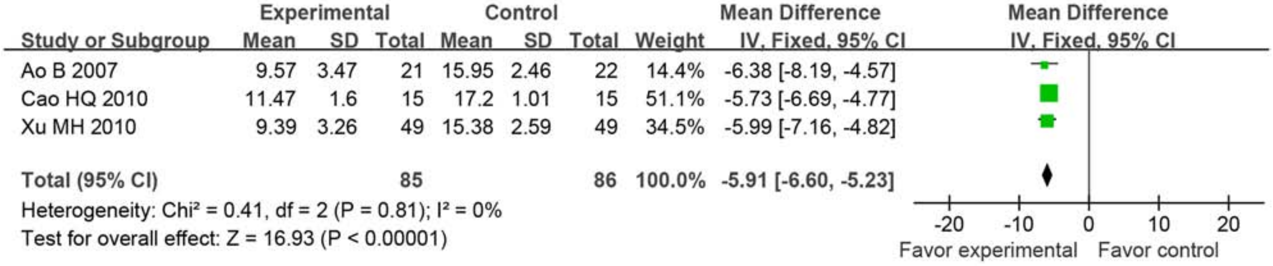

I. Gastrointestinal function and time to flatulence

Study or Subgroup	Experimental Mean	Experimental SD	Experimental Total	Control Mean	Control SD	Control Total	Weight	Mean Difference IV, Fixed, 95% CI
Chen XF 2008	52.12	7.57	26	58.35	7.66	26	38.2%	-6.23 [-10.37, -2.09]
You HH 2010	47.63	8.12	50	51.56	8.47	50	61.8%	-3.93 [-7.18, -0.68]
Total (95% CI)			76			76	100.0%	-4.81 [-7.37, -2.25]

Heterogeneity: Chi² = 0.73, df = 1 (P = 0.39); I² = 0%
Test for overall effect: Z = 3.68 (P = 0.0002)

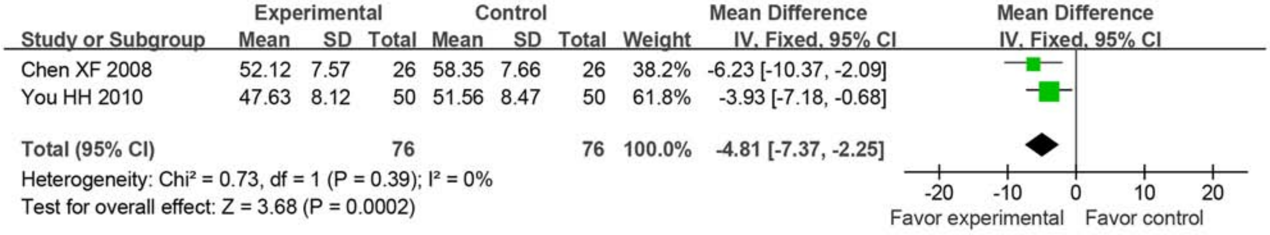

Supplementary Figure S2: Non-TCM PBIs improve quality of life in Chinese cancer patients.. PBIs interventions that evaluated overall quality of life (KPS[a],QLQ-C30[c] and QOL-CCC[b]), depression (SDS[d]), anxiety (SAS[e]), global mood (POMS-SF[f]), pain (VAS[g]), sleep (PSQI[h]) and time to flatulence were evaluated in a random effects statistical model. KPS[a] (Karnofsky Performance Score); QLQ-C30[b] (Quality of Life Questionnaire Core-30); QLQ-CCC[c] (Quality of Life Questionnaire for Chinese Cancer Patients with Chemobiotherapy); SDS[d] (Self-rating Depression scale); SAS[e] (Self-rating Anxiety scale); POMS-SF[f] (Profile of Mood States-short Form); VAS[g] (Visual analogue scale); PSQI[h] (Pittsburgh sleep quality index)

Supplementary Table S1: Study characteristics of meta-analysis data base for TCM PBIs

Author	Year	Total No. of patients	Type of cancer	Stage of tumor	Intervention type	Outcomes	Evaluating tools
Yu HX	2012	68	digestive organs	not report	acupuncture	post-surgery side effects and the function of intestines	Others
Pei Y	2010	67	breast	not report	acupuncture	depression	SDS SAS HAMA HAMD
Li HY	2008	45	digestive organs	not report	acupuncture	post-surgery side effects and function of intestines	Others
Liu H	2010	102	digestive organs	not report	acupuncture	pain	VAS
Feng Y	2011	80	digestive organs, respiratory and intrathoracic organs, lymphoid, haematopoietic, female genital organs and breast	not report	acupuncture	QoL, pain and depression	SDS HAMD PSQI
Yu L	2012	146	digestive organs, respiratory and intrathoracic organs	I-IV	acupuncture	chemo/ radiotherapy induced side effects and fatigue	Piper.PFS
Cheng L	2009	90	digestive organs, respiratory and intrathoracic organs, lip, oralcavity and pharynx and leukemia	not report	acupuncture	chemo/ radiotherapy induced side effects and diarrhea	Others
Chen CF	2012	36	digestive organs, respiratory and intrathoracic organs, lip, oralcavity and pharynx, female genital organs, leukemia and breast	not report	acupuncture	diarrhea	Others

(*continued*)

Author	Year	Total No. of patients	Type of cancer	Stage of tumor	Intervention type	Outcomes	Evaluating tools
Yang JL	2012	180	mixed malignant neoplasms	III-IV	acupuncture	chemo/ radiotherapy induced side effects and fatigue	KPS Piper. PFS
Xiang CY	2006	92	mixed malignant neoplasms	III-IV	acupuncture TCM FEMI TCM DS	QoL and depression	KPS SDS HAMD
Lai JH	2011	100	female genital organs	not report	massage	post-surgery side effects, depression and function of intestines	Others
Chen LR	2009	56	digestive organs	not report	massage	post-surgery side effects and pain	VAS
Wang HL	2012	399	digestive organs, respiratory and intrathoracic organs, lip, oralcavity and pharynx and breast	not report	massage	sleep and depression	SDS SAS PISQ
Chen YL	2008	400	digestive organs	I-IV	massage	post-surgery side effects and function of intestines	Others
Li ZY	2012	86	female genital organs	III-IV	dietary	QoL, chemo/ radiotherapy induced side effects and immune	KPS
Lu Y	2005	62	digestive organs, respiratory and intrathoracic organs, female genital organs, lymphoid, haematopoietic and breast	not report	acupuncture	QoL	KPS

Supplementary Table S2: Descriptive Summary of non-TCM-PBIs studies included in Meta-analysis

variable	Total sample (*n* = 51)		Music therapy (*n* = 20)		Relaxation training (*n* = 31)		Imaginary (*n* = 12)		Hypnosis (*n* = 1)		CBT (*n* = 22)			
	No.	%	No.	%	No.	%	No.	%	No.	%	No.	%	No.	%
**Population**														
Total No. of patients	5157		2119		3243		1664		43		1963		655	
median age, years	49		49		49		49		47		49		48	
females (%)	62		0		1		1		0		1		1	
**Type of cancer**														
mixed malignant neoplasms	15	29.41	6	11.76	8	15.69	3	5.88	0	0.00	8	15.69	2	3.92
digestive organs	12	23.53	8	15.69	4	7.84	2	3.92	0	0.00	4	7.84	1	1.96
respiratory and intrathoracic organs	10	19.61	6	11.76	5	9.80	3	5.88	0	0.00	5	9.80	2	3.92
lip, oral cavity and pharynx	2	3.92	1	1.96	2	3.92	1	1.96	0	0.00	2	3.92	1	1.96
breast	19	37.25	5	9.80	10	19.61	3	5.88	0	0.00	6	11.76	1	1.96
female genital organs	11	21.57	4	7.84	4	7.84	0	0.00	0	0.00	3	5.88	0	0.00
lymphoid, haematopoietic	1	1.96	1	1.96	0	0.00	0	0.00	0	0.00	0	0.00	0	0.00
bone and articular cartilage	1	1.96	0	0.00	0	0.00	0	0.00	0	0.00	0	0.00	0	0.00
male genital organs	0	0.00	0	0.00	0	0.00	0	0.00	0	0.00	0	0.00	0	0.00
eye, brain and other parts of central nervous system	1	1.96	1	1.96	1	1.96	1	1.96	0	0.00	1	1.96	0	0.00
urinary tract	6	11.76	4	7.84	4	7.84	3	5.88	0	0.00	4	7.84	1	1.96
thyroid and other endocrine glands	1	1.96	1	1.96	1	1.96	0	0.00	0	0.00	1	1.96	0	0.00
**Stage of tumor**														
I-II	7	13.73	0	0.00	2	3.92	0	0.00	0	0.00	0	0.00	0	0.00
III-IV	4	7.84	1	1.96	3	5.88	1	1.96	0	0.00	1	1.96	1	1.96
not reported	35	68.63	18	35.29	22	43.14	10	19.61	1	1.96	19	37.25	6	11.76
both	5	9.80	1	1.96	4	7.84	1	1.96	0	0.00	2	3.92	0	0.00

(*continued*)

variable	Total sample (*n* = 51)		Music therapy (*n* = 20)		Relaxation training (*n* = 31)		Imaginary (*n* = 12)		Hypnosis (*n* = 1)		CBT (*n* = 22)			
**Duration, days**														
median, days	34		3		33		22		60		30		29	
minimum	2		7		2		7		60		7		15	
maximum	168		84		168		60		60		84		60	
not reported	1		7		9		3		0		8		2	
**Outcomes**														
QoL score	27	52.94	12	23.53	14	27.45	8	15.69	1	1.96	13	25.49	7	13.73
appetite	4	7.84	1	1.96	1	1.96	1	1.96	0	0.00	1	1.96	1	1.96
sleepless	2	3.92	0	0.00	0	0.00	0	0.00	0	0.00	0	0.00	0	0.00
immunity	3	5.88	2	3.92	2	3.92	1	1.96	0	0.00	2	3.92	0	0.00
nausea and vomiting	7	13.73	3	5.88	3	5.88	1	1.96	0	0.00	3	5.88	1	1.96
thirst	0	0.00	0	0.00	0	0.00	0	0.00	0	0.00	0	0.00	0	0.00
abdominal distension	0	0.00	0	0.00	0	0.00	0	0.00	0	0.00	0	0.00	0	0.00
diarrhea	5	9.80	2	3.92	2	3.92	1	1.96	0	0.00	2	3.92	1	1.96
constipation	5	9.80	2	3.92	2	3.92	1	1.96	0	0.00	2	3.92	1	1.96
fatigue	6	11.76	3	5.88	3	5.88	1	1.96	0	0.00	3	5.88	1	1.96
radiation pneumonia	0	0.00	0	0.00	0	0.00	0	0.00	0	0.00	0	0.00	0	0.00
hair loss	0	0.00	0	0.00	0	0.00	0	0.00	0	0.00	0	0.00	0	0.00
hiccup	0	0.00	0	0.00	0	0.00	0	0.00	0	0.00	0	0.00	0	0.00
post-surgery lymph swelling	0	0.00	0	0.00	0	0.00	0	0.00	0	0.00	0	0.00	0	0.00
post-surgery pain	4	7.84	1	1.96	3	5.88	0	0.00	0	0.00	1	1.96	0	0.00
post-surgery insomnia	5	9.80	1	1.96	3	5.88	1	1.96	0	0.00	1	1.96	0	0.00
post-surgery immunology	0	0.00	0	0.00	0	0.00	0	0.00	0	0.00	0	0.00	0	0.00
post-surgery function of intestines	2	3.92	2	3.92	1	1.96	0	0.00	0	0.00	0	0.00	0	0.00
distress	2	3.92	11	21.57	19	37.25	5	9.80	0	0.00	13	25.49	1	1.96
pain induced by tumor	37	72.55	0	0.00	2	3.92	0	0.00	0	0.00	0	0.00	0	0.00
**Evaluating tools**														
KPS	3	5.88	3	5.88	3	5.88	3	5.88	1	1.96	3	5.88	3	5.88

(continued)

variable	Total sample (*n* = 51)		Music therapy (*n* = 20)		Relaxation training (*n* = 31)		Imaginary (*n* = 12)		Hypnosis (*n* = 1)		CBT (*n* = 22)			
QLQ-C30	10	19.61	5	9.80	6	11.76	3	5.88	0	0.00	5	9.80	3	5.88
QOL score without specific information	1	1.96	0	0.00	0	0.00	0	0.00	0	0.00	0	0.00	0	0.00
SCL90	2	3.92	0	0.00	1	1.96	0	0.00	0	0.00	0	0.00	0	0.00
SDS	20	39.22	5	9.80	9	17.65	4	7.84	0	0.00	6	11.76	0	0.00
SAS	27	52.94	8	15.69	14	27.45	3	5.88	0	0.00	10	19.61	0	0.00
HAMA	3	5.88	3	5.88	3	5.88	2	3.92	0	0.00	3	5.88	1	1.96
HAMD	2	3.92	2	3.92	2	3.92	2	3.92	0	0.00	2	3.92	1	1.96
VAS	7	13.73	2	3.92	5	9.80	0	0.00	0	0.00	2	3.92	0	0.00
PSQI	3	5.88	1	1.96	3	5.88	1	1.96	0	0.00	1	1.96	0	0.00
Other tools	25	49.02	13	25.49	0	0.00	6	11.76	1	1.96	14	27.45	4	7.84
**Publication types**														
journal articles	51	100.00	20	39.22	31	60.78	12	23.53	1	1.96	22	43.14	7	13.73
conference proceedings	0	0.00	0	0.00	0	0.00	0	0.00	0	0.00	0	0.00	0	0.00
dissertations	0	0.00	0	0.00	0	0.00	0	0.00	0	0.00	0	0.00	0	0.00
**Publication year**														
2013	5	9.80	0	0.00	0	0.00	0	0.00	0	0.00	0	0.00	0	0.00
2012	7	13.73	0	0.00	0	0.00	0	0.00	0	0.00	0	0.00	0	0.00
2011	9	17.65	0	0.00	1	1.96	0	0.00	0	0.00	0	0.00	0	0.00
2010	8	15.69	0	0.00	8	15.69	0	0.00	0	0.00	0	0.00	0	0.00
2009	5	9.80	3	5.88	5	9.80	0	0.00	0	0.00	5	9.80	0	0.00
2008	5	9.80	5	9.80	5	9.80	0	0.00	0	0.00	5	9.80	0	0.00
2007	4	7.84	4	7.84	4	7.84	4	7.84	0	0.00	4	7.84	0	0.00
2006	5	9.80	5	9.80	5	9.80	5	9.80	0	0.00	5	9.80	4	7.84
2005	1	1.96	1	1.96	1	1.96	1	1.96	0	0.00	1	1.96	1	1.96
2004	1	1.96	1	1.96	1	1.96	1	1.96	0	0.00	1	1.96	1	1.96
2003	0	0.00	0	0.00	0	0.00	0	0.00	0	0.00	0	0.00	0	0.00
2002	0	0.00	0	0.00	0	0.00	0	0.00	0	0.00	0	0.00	0	0.00
2001	1	1.96	1	1.96	1	1.96	1	1.96	1	1.96	1	1.96	1	1.96
2000	0	0.00	0	0.00	0	0.00	0	0.00	0	0.00	0	0.00	0	0.00
Total No. of publications	51	100.00	20	39.22	31	60.78	12	23.53	1	1.96	22	43.14	7	13.73

Supplementary Table S3: Study characteristics of meta-analysis data base for non-TCM PBIs

Author	Year	Total No. of patients	Type of cancer	Stage of tumor	Intervention type	Outcomes	Evaluating tools
Zhang H	2009	92	not reported	not reported	CBT, relaxation training, music therapy, imaginary therapy	QOL, chemo/ radiotherapy induced side effects	QLQ-C30 POMS-SF
Li HM	2007	101	digestive, respiratory and intrathoracic, lymphoid, haematopoietic, bone and articular cartilage, urinary tract organs	not reported	music therapy	depression, anxiety	SDS SAS
Wang JP	2006	408	not reported	not reported	relaxation and imaginary therapy	QOL	QLQ-C30
Jia HZ	2011	70	breast	III-IV	relaxation training	pain, anxiety, function of intestines	SAS VAS
Peng HZ	2012	52	breast	not reported	relaxation and imaginary therapy	post-surgery side effects, depression, anxiety	SDS SAS
Xiong FZ	2009	80	digestive, respiratory and intrathoracic, breast, thyroid organs	not reported	relaxation training	post-surgery side effects, anxiety	SAS
Chen XF	2008	52	digestive organs	not reported	relaxation training and music therapy	pain	VAS
Xu MH	2010	98	female genital organs	I-II	relaxation training and music therapy	sleep	PSQI
Long XL	2011	202	female genital organs	not reported	relaxation training	chemo/ radiotherapy induced side effects, anxiety	SAS
Niu X	2013	70	digestive, respiratory and intrathoracic, breast, female genital organs, lymphoid and haematopoietic, thyroid organs	I-II	CBT and relaxation training	pain, anxiety	SAS VAS

Author	Year	Total No. of patients	Type of cancer	Stage of tumor	Intervention type	Outcomes	Evaluating tools
Jiang JF	2008	104	respiratory and intrathoracic, breast organs	not reported	relaxation training, music therapy, imaginary therapy	QOL, chemo/ radiotherapy induced side effects, anxiety	SAS
Yao YY	2009	80	breast and female genital organs	not reported	CBT	QOL, chemo/ radiotherapy induced side effects, pain	QLQ-C30 VAS
Cao HQ	2010	30	digestive organs	III-IV	relaxation training	post-surgery side effects, pain	VAS PSQI
Wang WL	2013	206	female genital organs	III-IV	relaxation training	QOL chemo/ radiotherapy induced side effects, depression	QLQ-C30 SDS
Ao B	2007	43	eye, brain and other parts of central nervous system	not reported	CBT, relaxation training and hypnosis	sleep	PSQI
Li Y	2012	42	urinary tract	not reported	CBT and relaxation training	QOL, post-surgery side effects, pain, depression, anxiety	QOL SDS SAS
Wang YE	2012	80	female genital organs	I-II	CBT	QOL, post-surgery side effects, depression, anxiety	SDS SAS
Ye LX	2013	125	digestive organs	not reported	CBT	QOL, post-surgery side effects, depression, anxiety	QLQ-C30 SDS SAS
Hao YJ	2011	60	digestive organs	not reported	CBT and relaxation training	QOL, post-surgery side effects, depression	SDS SAS SF-36
Bai SM	2004	45	lip, oralcavity and pharynx	not reported	CBT and relaxation training	QOL	QLQ-C30
Wen SM	2009	113	lip, oralcavity and pharynx	III-IV	relaxation training and music therapy, psychotherapy	QOL, depression, anxiety	SDS SAS WHOQOL

(*continued*)

Author	Year	Total No. of patients	Type of cancer	Stage of tumor	Intervention type	Outcomes	Evaluating tools
Li XL	2012	96	breast	I-II	psychotherapy	QOL, depression, anxiety	SDS SAS FACT
Xu L	2012	99	not reported	not reported	music therapy	QOL, chemo/radiotherapy induced side effects and pain	EORTC-QLQ-B223
Cheng K	2006	30	digestive, respiratory and intrathoracic, breast, urinary tract organs	III-IV	CBT and relaxation training, psychotherapy	QOL, depression, anxiety	QLQ-C30 HAMA HAMD POMS
Tian XY	2012	80	not reported	I-II	CBT	QOL and depression	QLQ-C30 SCL90
Cao DL	2006	90	digestive organs	not reported	CBT, relaxation training, music therapy, imaginary therapy	QOL	KPS
Xie Z	2006	175	respiratory and intrathoracic	not reported	CBT, relaxation training, music therapy, imaginary therapy	QOL	KPS
Cui JF	2007	60	breast	not reported	CBT	post-surgery side effects, depression, anxiety	SDS SAS
Chen JZ	2011	80	breast	I-II	CBT	QOL, post-surgery side effects, depression, anxiety	SDS SAS
Zhang MF	2008	120	breast	not reported	CBT and relaxation training	QOL, depression, anxiety	SDS SAS
Mu SY	2010	80	female genital organs	III-IV	CBT and relaxation training	pain, depression, anxiety	SAS VAS
Chen JJ	2011	66	digestive organs	not reported	relaxation and imaginary therapy	QOL, post-surgery side effects, chemo/radiotherapy induced side effects, pain, depression	QLQ-C30

(*continued*)

Author	Year	Total No. of patients	Type of cancer	Stage of tumor	Intervention type	Outcomes	Evaluating tools
Li CM	2012	110	breast	not reported	CBT, relaxation training, imaginary therapy	QOL, post-surgery side effects, chemo/ radiotherapy induced side effects, depression	QLQ-C30
Lin XY	2010	97	breast	III-IV	CBT, relaxation training, imaginary therapy	post-surgery side effects, chemo/ radiotherapy induced side effects, depression, anxiety	SDS SAS
Lu FM	2013	130	breast	not reported	CBT, relaxation training, imaginary therapy	post-surgery side effects, depression, anxiety	SDS SAS
Li L	2011	80	breast	I-II	relaxation training, imaginary therapy	chemo/ radiotherapy induced side effects, depression, anxiety	SDS SAS
Liu AM	2006	111	digestive, respiratory and intrathoracic, female genital, urinary tract organs	not reported	music therapy	post-surgery side effects, depression	SDS STAI
You HH	2010	100	female genital organs	I-II	music therapy	post-surgery side effects, function of intestines	Prince-Aerny
Zhang WZ	2010	107	digestive, respiratory and intrathoracic, breast organs	not reported	music therapy	chemo/ radiotherapy induced side effects and depression	SDS SAS
Wang J	2009	60	female genital organs	not reported	music therapy	chemo/ radiotherapy induced side effects, anxiety	SAS

(*continued*)

Author	Year	Total No. of patients	Type of cancer	Stage of tumor	Intervention type	Outcomes	Evaluating tools
Zhou KN	2010	120	breast	not report	music therapy	depression	SDS
Yang XH	2008	80	digestive organs	not reported		post-surgery side effects, anxiety	SAS STAI
Zhao PT	2008	95	digestive, respiratory and intrathoracic, breast, female genital, urinary tract organs	not reported	music therapy	chemo/ radiotherapy induced side effects, anxiety	SAS HAMA
Cao GR	2007	82	digestive organs	III-IV	music therapy	QOL, chemo/ radiotherapy induced side effects, depression, anxiety	SDS SAS HAMA HAMD
Bi HW	2011	60	digestive organs	not reported	music therapy	anxiety	SAS
Xie Z	2001	260	not reported	not reported	relaxation training, music therapy, imaginary therapy	QOL, depression	KPS
Le GA	2005	64	lymphoid, haematopoietic	not reported	relaxation training, psychotherapy	QOL, chemo/ radiotherapy induced side effects, pain	QLQ-C30
Tian JM	2010	96	lymphoid, haematopoietic	not reported	CBT and relaxation training, psychotherapy	QOL, depression	QLQ-C30 SCL90
Mei JH	2011	78	lymphoid, haematopoietic	not reported	relaxation training, psychotherapy	QOL, depression, anxiety	SDS SAS
Li XM	2011	120	breast	not reported	music therapy	depression	SDS
Guo Z	2013	178	respiratory and intrathoracic, breast and female genital organs	not reported	CBT, psychotherapy	QOL, chemo/ radiotherapy induced side effects, depression, anxiety	SDS SAS
